# Human Anatomy

**Published:** 1898-06

**Authors:** 


**Human Anatomy.** By John Cleland, M.D., F.R.S., and John Yule Mackay, M.D. Pp. xx., 833. Glasgow: James MacLehose & Sons. 1896.

We looked forward with some interest to the publication of this book; for, coming from such a school and written by such authors, everything augured in its favour. We are afraid we expected too much; at all events, we thought that this book would be a distinct improvement on such works as Morris and the classic Gray, but we are bound to express disappointment. There is perhaps no section in the book which excels any corresponding one in the works alluded to; indeed, we think there is not one which equals the others. As a record of Professor Cleland's work the book is without doubt of interest to the teacher; but we doubt if the student, whose mind is occupied rather with the problem of passing examinations, will appreciate much of the very interesting personal work mentioned here.

We do not propose to criticise each section, but we would express the opinion that those dealing with the bones and with the abdominal viscera are distinctly below the level one could reasonably expect in such a work. As to the section on bones, we may say that it is exceedingly difficult to read, and so wanting in good plates that the junior student must find himself in a hopeless state and in sheer desperation resort to some other work; and in speaking of good plates, drawings, etc., we cannot refrain from saying that the majority of the original illustrations are at least disappointing—many of them, even to the practised eye, being almost undecipherable, though to the same eye they give evidence of representing most excellent preparations, and they shew many original views of situations. They are a very sorry contrast to the magnificent illustrations borrowed from Testut.

In the section dealing with the abdominal viscera we notice with regret that the later ideas as to the supposed true shape of the viscera are practically ignored. This we think is a grievous omission; for whatever be the intrinsic merits of the views at present held by the majority of anatomists concerning the viscera, the fact that such views are held by such men at least warrants that they should have a place in a book which is apparently a student's text-book.

